# Fascial Plane Blocks for Analgesia in Non-Operating Room Anesthesia Settings

**DOI:** 10.3390/jcm15062143

**Published:** 2026-03-11

**Authors:** Huseyin Ulas Pinar, Asina Pinar, Ayşe Heves Karagöz

**Affiliations:** 1 Department of Anesthesiology and Reanimation, Hacettepe University School of Medicine, Ankara 06230, Türkiye; 2 Department of Anesthesiology and Reanimation, Ankara Bilkent City Hospital, University of Health Sciences, Ankara 06800, Türkiye

**Keywords:** non-operating room anesthesia, fascial plane blocks, regional anesthesia, opioid-sparing analgesia, interventional cardiology, interventional radiology

## Abstract

Non-operating room anesthesia (NORA) has emerged as one of the fastest-growing domains of modern anesthetic practice. Increasing procedural complexity and an aging, comorbid patient population demand analgesic strategies that enhance safety, comfort, and procedural success while minimizing physiological disturbance. Although systemic opioids and sedatives remain commonly used in NORA settings, their dose-dependent adverse effects may compromise patient safety and delay recovery, particularly in environments with limited postprocedural monitoring. Ultrasound-guided fascial plane blocks (FPBs) have therefore gained prominence as key components of opioid-sparing and opioid-free anesthetic strategies. By providing targeted regional analgesia with preserved hemodynamic stability, FPBs reduce systemic analgesic requirements and opioid-related side effects while improving patient comfort. This review summarizes the anatomical basis, proposed mechanisms of action, and current clinical evidence supporting the use of thoracic and abdominal fascial plane blocks in NORA settings, with particular emphasis on interventional cardiology and interventional radiology procedures. The expanding role of FPBs suggests that these techniques may become integral elements of standard analgesic protocols in contemporary non-operating room anesthesia practice.

## 1. Introduction

Non-operating room anesthesia (NORA) represents one of the most dynamic and rapidly expanding fields in modern medicine. As the complexity of diagnostic and therapeutic interventional procedures continues to increase, the profile of patients undergoing these procedures has undergone a substantial transformation. Currently, patients referred to NORA settings are frequently of advanced age, burdened with multiple comorbidities—such as heart failure, chronic obstructive pulmonary disease, and obesity—and often have markedly reduced physiological reserves. In this patient population, the goals of anesthetic management extend beyond achieving technical procedural success to include the optimization of perioperative comfort and safety. This consideration becomes even more critical in non-operating room environments, where equipment availability and personnel support may be more limited compared with conventional operating room settings [1,2,3].

In this fragile patient population, establishing a safe and effective analgesic plan is essential to reduce cardiopulmonary complications, achieve early discharge targets in ambulatory patients, and prevent unplanned hospital readmissions resulting from inadequate pain control. Traditionally, systemic opioids and sedative agents—such as propofol, midazolam, and remifentanil—have been used for pain management during these procedures. However, the use of systemic opioids for analgesia is associated with a range of dose-dependent adverse effects, including respiratory depression, hemodynamic instability, nausea and vomiting, ileus, urinary retention, and cognitive dysfunction. In non-operating room anesthesia environments, where monitoring capabilities and postoperative care resources may be limited, these adverse effects may pose significant risks to patient safety and lead to delayed discharge [4].

In this context, regional analgesic techniques—particularly ultrasound-guided fascial plane blocks (FPBs)—have become key components of opioid-sparing or opioid-free anesthetic strategies. By providing targeted analgesia, fascial plane blocks enhance patient comfort and safety, minimize the need for systemic analgesics, and thereby reduce the incidence of opioid-related adverse effects [5].

In recent years, the routine integration of ultrasonography into anesthetic practice, together with cadaveric studies and dye distribution analyses using radiological imaging modalities such as magnetic resonance imaging (MRI) and computed tomography (CT), has fundamentally transformed the understanding and clinical application of fascial plane blocks. The principal factors contributing to the growing popularity of these techniques include the following:

(a) Ease of Application and Learning Curve:

Compared with traditional neuraxial techniques (such as epidural and spinal anesthesia) or deep plexus blocks, fascial plane blocks can be performed using more superficial anatomical landmarks and generally exhibit a steeper learning curve. The ability to easily identify relevant anatomical structures with ultrasonography has facilitated the widespread adoption of these techniques in clinical practice.

(b) Safety Profile:

Because major vascular and neural structures—such as the neuraxis, pleura, or large blood vessels—are not directly targeted, and instead the fascial layers surrounding muscle groups are used as the injection plane, fascial plane blocks demonstrate a relatively favorable safety profile. These characteristics make them particularly attractive alternatives to neuraxial techniques, especially in patients receiving anticoagulant or antiplatelet therapy [6].

(c) Hemodynamic Stability:

The absence or minimal extent of sympathetic blockade compared with neuraxial anesthesia provides a significant advantage in patients with limited hemodynamic reserve or an increased risk of cardiovascular instability.

(d) Guideline Recommendations:

Recently published international guidelines recommend fascial plane blocks as robust components of multimodal analgesia for a wide range of surgical and interventional procedures [5,7,8,9,10].

### Definition and Mechanisms of Action of Fascial Plane Blocks

FPBs refer to a group of regional anesthesia techniques in which relatively large volumes of local anesthetic are injected into interfascial spaces surrounding muscle groups, without directly targeting peripheral nerves or nerve plexuses. The primary objective of these techniques is to facilitate the passive spread of the local anesthetic along the fascial plane (hydrodissection), allowing the drug to reach neural structures or nerve terminals traversing these compartments. In contrast to conventional nerve blocks—such as the femoral nerve block—where the nerve itself is the direct target, FPBs aim to anesthetize a broader anatomical “field” or “compartment.”

Although the exact mechanisms underlying the analgesic effects of FPBs have not been fully elucidated, several complementary mechanisms have been proposed in the literature and are thought to act synergistically:

(a) Bulk Flow

This mechanism refers to the longitudinal and transverse spread of the local anesthetic along the fascial plane, driven by gravity and tissue resistance, enabling the drug to reach adjacent anatomical compartments through which the target nerves course. The extent of this spread is directly related to the volume of local anesthetic injected.

(b) Transfascial Spread and Microscopic Diffusion

Local anesthetic may spread to adjacent compartments and deeper neural structures—such as the paravertebral space or epidural space—either through macroscopic fascial openings (e.g., those traversed by neurovascular bundles) or via microscopic diffusion across the inherently permeable fascial tissue. This mechanism is particularly relevant in techniques such as the erector spinae plane (ESP) block, where ventral and dorsal rami involvement has been attributed to such diffusion pathways.

(c) Systemic Absorption

Fascial planes often encompass large surface areas and are highly vascularized, particularly in regions surrounding muscle groups. Systemic absorption of local anesthetics from these planes may contribute to analgesic, anti-inflammatory, and antihyperalgesic effects analogous to those observed with intravenous lidocaine infusion. This mechanism may partly explain the broader-than-expected analgesic effects associated with certain fascial plane blocks [11].

In this review, FPBs applied in NORA settings—such as interventional cardiology and interventional radiology—are classified according to their anatomical regions and clinical indications. Their clinical applications and outcomes are discussed in light of the current literature, without an in-depth focus on technical procedural details. Broadly, these blocks can be categorized into two main groups: thoracic and abdominal truncal blocks.

## 2. Search Strategy

This narrative review was conducted using a structured but non-systematic literature search strategy. Electronic databases, including PubMed/MEDLINE, Embase, and Google Scholar, were searched for articles published between January 2015 and January 2026. Keywords included “fascial plane block”, “non-operating room anesthesia”, “NORA”, “interventional radiology anesthesia”, “interventional cardiology anesthesia”, “erector spinae plane block”, “quadratus lumborum block”, and “local anesthetic systemic toxicity”. Randomized controlled trials, prospective and retrospective observational studies, systematic reviews, and relevant case series were included. Case reports were included when higher-level evidence was unavailable, particularly in emerging NORA applications. Pediatric and adult populations were both considered. The PRISMA methodology was not formally applied due to the narrative design; however, study selection prioritized methodological rigor and clinical relevance.

## 3. Thoracic Fascial Plane Blocks

Interventional procedures involving the thoracic region pose a significant challenge for anesthesiologists in terms of pain management, owing to the complex and multilayered innervation of the chest wall. The effectiveness of ultrasound-guided fascial plane blocks in this region relies heavily on a comprehensive and detailed understanding of thoracic anatomy. For successful analgesia, precise concordance is required between the procedural field, the affected anatomical structures—such as skin, muscle, bone, and pleura—and the dermatomal and osteotomal coverage provided by the selected block. In many cases, blockade of cutaneous innervation alone is insufficient; adequate analgesia may also require effective coverage of the innervation of muscles, ligaments, joints, and osseous structures, including the ribs and sternum.

### Anatomy and Innervation of the Thoracic Wall

The anterolateral chest wall consists of superficial muscular layers (pectoralis major and minor anteriorly, serratus anterior laterally, and latissimus dorsi posterolaterally) overlying the intercostal muscles and ribs. The principal sensory innervation derives from the intercostal nerves (T2–T12), which course within the intercostal spaces and give rise to lateral and anterior cutaneous branches. These branches are the primary targets of most thoracic fascial plane blocks.

In addition to intercostal innervation, the pectoral nerves (from the brachial plexus), the long thoracic nerve, and the thoracodorsal nerve contribute motor and partial sensory supply to the pectoral and serratus muscle compartments. Cutaneous innervation of the upper anterior chest is further supplemented by the supraclavicular nerves (C3–C4) from the cervical plexus. Consequently, sensory input to the anterior chest wall originates from three distinct sources: the thoracic spinal nerves, the brachial plexus, and the cervical plexus.

From a block-oriented perspective, the chest wall can be divided into three clinically relevant regions:

a. Lateral Thoracic Region

Innervated predominantly by the lateral cutaneous branches of the intercostal nerves (T2–T9). This region is effectively targeted by the serratus anterior plane (SAP) block or pectoserratus (PECS II) block.

b. Parasternal (Anteromedial) Region

Primarily supplied by the anterior cutaneous branches of the T2–T6 intercostal nerves. These branches traverse the parasternal intercostal spaces and can be targeted via transversus thoracic muscle plane or parasternal intercostal plane blocks.

c. Posterior Thoracic Region

Innervated by the dorsal rami of the thoracic spinal nerves. Blocks performed in the paraspinal interfascial planes—most notably the ESP block and retrolaminar block—aim to achieve multisegmental spread with potential anterior extension toward the paravertebral space.

**Figure 1:**
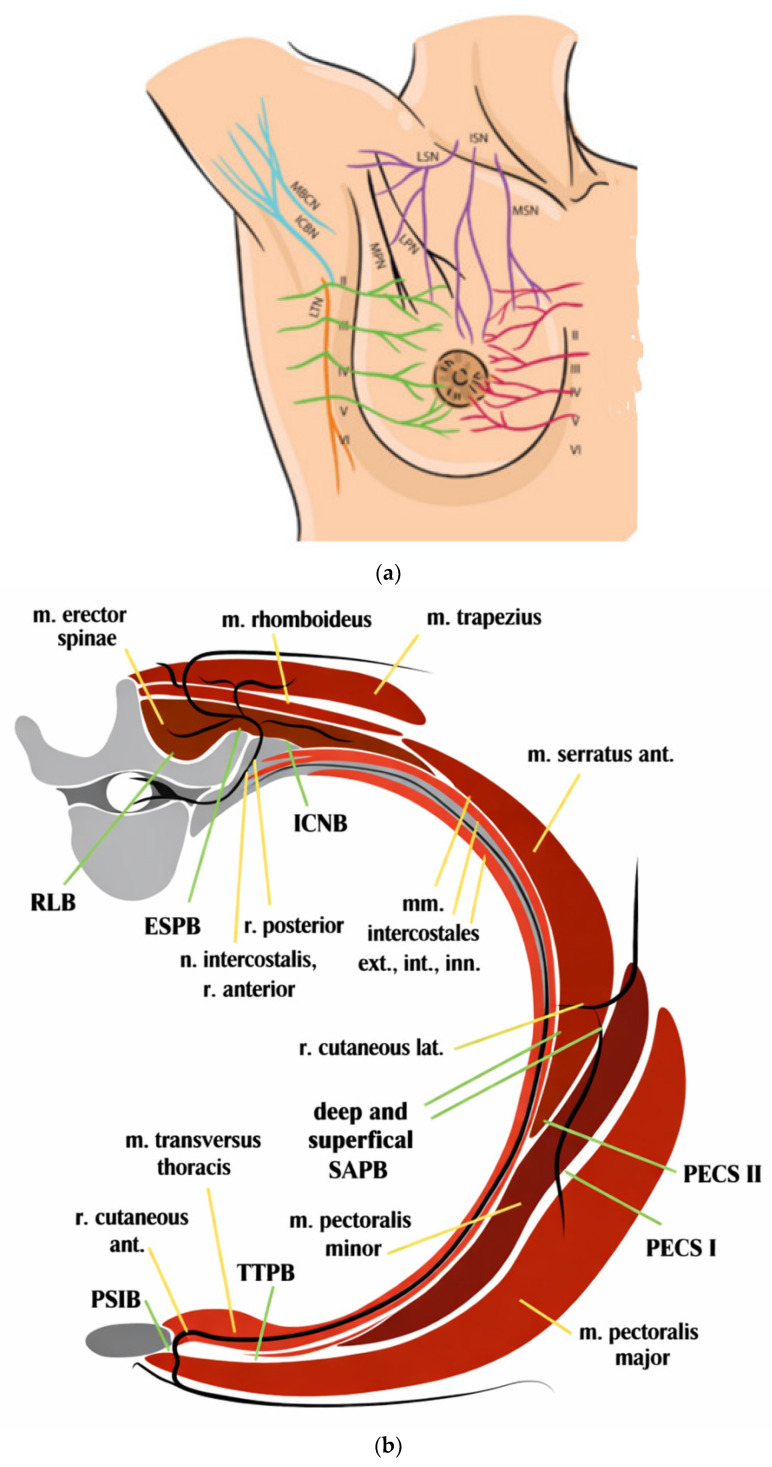
Innervation of the thoracic wall and fascial plane blocks. (**a**). Innervation of the thoracic wall; LTN: long thoracic nerve; LSN: lateral supraclavicular nerve; ISN: intermediate supraclavicular nerve; MSN: medial supraclavicular nerve; LPN: lateral pectoral nerve; MPN: medial pectoral nerve; II: anterior cutaneous branch of the second intercostal nerve; III–VI (green): lateral cutaneous branches of the thoracic intercostal nerves; II–VI (red): anterior cutaneous branches of the thoracic intercostal nerves; ICBN: intercostobrachial nerve; MBCN: medial brachial cutaneous nerve. (**b**) Thoracic fascial plane blocks on a cross-section of the thoracic wall. RLB: Retrolaminar block; ESPB: Erector spinae plane block; ICNB: Intercostal nerve block; SAPB: Serratus anterior plane block; PECS I: Interpectoral block; PECS II: Pectoserratus block; TTPB: Transversus thoracis plane block; PSIB: Parasternal intercostal plane block.

Understanding these compartment-based innervation patterns is essential in NORA procedures, where tailored combinations of FPBs may be required to match the dermatomal distribution of procedural pain while minimizing systemic analgesic requirements [12] (Figure 1).

A summary of thoracic fascial plane blocks, including their indications and technical characteristics, is provided in Table 1.

## 4. Abdominal Fascial Plane Blocks

The abdominal wall is composed of multiple muscle groups surrounding the peritoneum, interconnected by aponeurotic structures. The nerves responsible for sensory innervation of the abdominal region course within the fascial planes between these muscle layers. Ultrasound-guided blocks provide somatic analgesia over a wide area—and, in certain techniques, visceral analgesia—by depositing local anesthetic within these fascial planes.

### Anatomy and Innervation of the Abdominal Wall

In the context of FPBs for NORA, the abdominal wall should be considered as a layered neuromuscular compartment in which sensory nerves travel within predictable interfascial planes that are amenable to ultrasound-guided blockade.

The anterior abdominal wall is primarily innervated by the anterior rami of the T6–L1 spinal nerves, including the intercostal, subcostal, iliohypogastric, and ilioinguinal nerves. These nerves course from lateral to medial within the fascial plane between the internal oblique and transversus abdominis muscles—commonly referred to as the transversus abdominis plane (TAP)—before entering the rectus sheath and terminating as anterior cutaneous branches.

**Figure 2:**
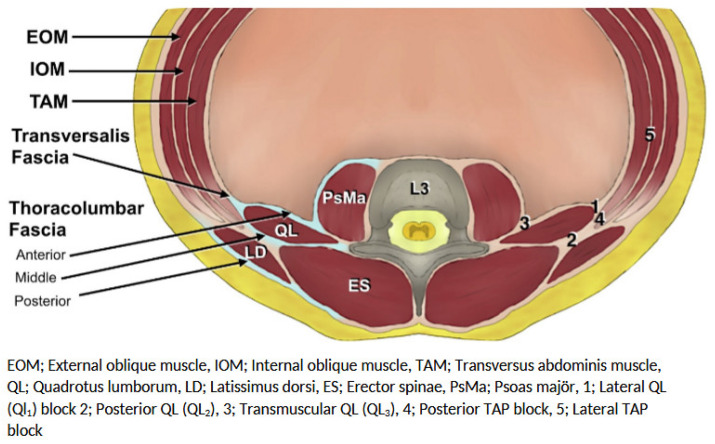
Abdominal fascial plane blocks.

From a practical block-selection perspective, the abdominal wall may be divided into three clinically relevant regions:

a. Anterolateral Abdominal Wall
  This area is characterized by somatic innervation from T6–L1 traveling within the transversus abdominis plane. This region is effectively targeted by the TAP block, subcostal TAP block (for supraumbilical coverage), and ilioinguinal–iliohypogastric block (for lower abdominal and groin procedures). These techniques provide reliable somatic analgesia but limited visceral pain control.

b. Midline (Periumbilical and Epigastric) Region
  This area is innervated by the anterior cutaneous branches that traverse the rectus sheath. The rectus sheath block specifically targets these branches and is particularly useful in procedures involving midline access or periumbilical interventions.

c. Posterior Abdominal Wall and Paraspinal Region
  The quadratus lumborum (QL) muscle, psoas major muscle, and thoracolumbar fascia form key anatomical landmarks for deeper interfascial blocks. QL and abdominal ESP blocks are performed in these posterior fascial compartments and may allow cranio-caudal spread with potential paravertebral or sympathetic chain involvement.
  This posterior approach is particularly relevant in NORA procedures involving visceral manipulation (e.g., hepatic ablation or biliary drainage), where broader dermatomal coverage and possible visceral analgesia are desirable.
  This compartment-based anatomical framework facilitates rational block selection tailored to the expected pain distribution of the specific NORA procedure while minimizing systemic opioid requirements [13] (Figure 2). A summary of abdominal FPBs, including their indications and technical characteristics, is presented in Table 2.

## 5. Interventional Cardiology

In the field of interventional cardiology, pain management is a critical issue, particularly during the implantation of cardiac devices. Although patients are commonly managed under sedation, significant pain may occur during surgical incision, pectoral muscle dissection, creation of the device pocket, and tunneling of the leads.

### 5.1. Subcutaneous Implantable Cardioverter–Defibrillator (S-ICD) Implantation

S-ICD implantation is a technology developed as an alternative to transvenous systems and does not require intracardiac or intravascular lead placement. The procedure involves a thoracic wall incision in the midaxillary region for placement of the pulse generator, followed by intermuscular dissection and creation of a subcutaneous tunnel in the parasternal region for positioning of the defibrillator lead. This process generates extensive somatic nociceptive input covering the T3–T9 dermatomes and is generally more painful than standard transvenous ICD implantation.

To reduce the need for deep sedation, fascial plane blocks may be utilized. Zhang et al. [14] investigated the efficacy of a combination of SAP and transversus thoracic muscle plane (TTMP) blocks as an adjunct to local anesthesia in patients undergoing S-ICD implantation. In this randomized controlled trial, sedoanalgesia was provided using dexmedetomidine and remifentanil infusions. Compared with the local anesthesia-only group, the regional anesthesia group demonstrated significantly lower intraprocedural pain scores, reduced sedative requirements (dexmedetomidine and remifentanil), and decreased postoperative pain levels. In addition, the incidence of postoperative nausea and vomiting and hypoxemia was lower in the block group [14].

In obese patients with reduced ejection fraction (<35%), an SAP block administered 30 min before the procedure was shown to maintain significantly lower Numeric Rating Scale (NRS) scores (NRS < 3) during the procedure and for up to 12 h postoperatively [15].

Szamborski et al. [16] described a combination of PECS II and superficial SAP blocks for S-ICD implantation in high-risk patients (ASA III–IV). In this single-center study conducted in Poland, a series of 16 patients underwent the procedure under sedation using this block combination. The authors reported that it provided a safe and comfortable surgical environment without causing hemodynamic instability. The mean local anesthetic volumes were 19.4 mL for the PECS II block and 34.7 mL for the SAP block, and 50% of patients did not require any additional non-opioid analgesics. The authors suggested this technique as a safe alternative for patients who are unable to tolerate general anesthesia [16].

### 5.2. Implantable Cardioverter–Defibrillator (ICD) and Pacemaker Implantation

Most ICD and pacemaker implantations involve the creation of a device pocket in the left pectoral region through the deltopectoral groove, along with axillary or subclavian venous puncture. The innervation of the surgical field is provided by the supraclavicular nerves (cutaneous innervation of the pocket area) and the pectoral nerves (deep tissues and muscles of the pocket).

PECS I and PECS II blocks provide analgesia of the pectoral muscles and axillary region and have been shown to reduce postoperative pain in these procedures. In a multicenter study published in 2025 that included 602 patients, PECS I and PECS II blocks, administered either alone or in combination, provided superior postoperative analgesia and reduced opioid consumption compared with standard subcutaneous local anesthesia (4-h VAS score: 1.5 ± 2.1 vs. 4.5 ± 2.5, *p* < 0.001) [17]. The efficacy of these blocks has also been demonstrated in pediatric patients undergoing ICD implantation [18].

The clavipectoral fascial plane block (CPFB), originally described for clavicular fractures, has recently emerged as a promising technique for providing analgesia of the clavicular and pectoral regions during pacemaker and ICD implantation. CPFB targets the nerve branches innervating the clavicular periosteum and surrounding tissues, including the supraclavicular nerves, the nerve to subclavius, and the pectoral nerves. Metinyurt and Sandeep reported successful permanent pacemaker implantation in high-risk patients using CPFB with minimal sedation or opioid administration. These case reports suggest that CPFB may serve as a potential “stand-alone anesthesia” technique for device implantation procedures without sedation [19,20].

## 6. Interventional Radiology

In interventional radiology units, there is an increasing need for fascial plane blocks, particularly during non-vascular procedures. Ablative treatments targeting organs such as the liver, kidney, and lung, as well as biliary drainage procedures, may cause severe somatic and visceral pain due to capsular distension, pleural irritation, and traversal of muscular planes.

### 6.1. Hepatic Tumor Ablation and Biliary Drainage

Microwave ablation or radiofrequency ablation (RFA) of hepatic lesions often results in severe pain, especially when tumors are subcapsular or adjacent to the diaphragm. In addition, percutaneous transhepatic biliary drainage (PTBD) is a painful procedure because it requires passage through the liver parenchyma and costal structures, and it frequently necessitates deep sedation and/or opioid administration. Traditionally, techniques such as paravertebral block, celiac plexus block, or hepatic hilar block have been employed; however, fascial plane blocks have gained prominence due to their less invasive nature.

The ESP block has emerged as a promising and versatile technique for abdominal organ interventions, owing to its ability to provide both somatic (thoracic and abdominal wall) and visceral analgesia through involvement of the sympathetic chain. In a retrospective comparative study published in 2025, Erdim et al. [21] evaluated 101 patients undergoing PTBD and compared preprocedural bilateral ESP block performed at the T8 level with standard procedural fentanyl analgesia. Median Numeric Rating Scale (NRS) pain scores at the 1st, 2nd, and 6th postoperative hours were significantly lower in the ESP block group than in the sedation-only group (NRS at 1 h: 3 vs. 6; *p* < 0.001) [21]. The authors concluded that the ESP block provides effective analgesia for PTBD, minimizes opioid consumption, enhances patient comfort, and contributes to procedural success.

Recent studies have also demonstrated that ESP block alone can provide sufficient analgesia for hepatic microwave ablation without the need for deep sedation or general anesthesia. Lucatelli et al. [22] reported in a series of 54 patients that ESP block performed at the T6–T9 levels allowed the completion of the procedure without additional sedation in 74% of cases. This approach enabled patients to remain awake and cooperate with breath-holding commands during diaphragmatic motion, thereby improving procedural accuracy and success [22].

In situations where a single block may be insufficient, combined techniques have been proposed. For example, the combination of a TAP block and a hepatic hilar block has been shown to provide superior analgesia for hepatic interventions compared with either the TAP block or the hepatic hilar block alone [23].

### 6.2. Vascular and Neurovascular Interventions

Although the literature on the use of fascial plane blocks in vascular and neurovascular interventions remains relatively limited, their application for access-site analgesia is increasing. In procedures such as transarterial chemotherapy or chemoembolization for hepatic malignancies, visceral pain predominates, and central techniques such as paravertebral blocks may be more effective. Nevertheless, fascial plane blocks play a valuable role in managing access-site pain.

In procedures such as endovascular aortic repair (EVAR), thoracic endovascular aortic repair (TEVAR), and transcatheter aortic valve implantation (TAVI), the primary access route is typically the femoral artery via the inguinal region. Placement of large-bore sheaths and the use of arterial closure devices can cause significant pain. For femoral artery access in the inguinal region, the fascia iliaca block is the most commonly used technique. By blocking the femoral nerve, the lateral femoral cutaneous nerve, and the obturator nerve with a single injection, the fascia iliaca block provides excellent analgesia of the anterior and lateral thigh as well as the groin region.

Case reports in the literature have described successful emergency femoral thrombectomy or EVAR performed solely under fascia iliaca block in patients at high risk for general or neuraxial anesthesia (e.g., those with recent acute myocardial infarction) [24,25]. Additionally, Kılıçaslan et al. described the fascia lata plane block as a novel ultrasound-guided technique for percutaneous endovascular procedures and demonstrated its effectiveness in controlling lateral thigh pain [26].

During the placement of tunneled central venous catheters (e.g., Hickman catheters or implantable ports), PECS I and PECS II blocks may be combined with cervical plexus blocks to provide effective analgesia for pocket creation and subcutaneous tunneling in the pectoral region [27].

## 7. Emergency Department

Moderate-to-severe pain is a common complaint in the emergency department. FSB applications have been increasingly used in this setting due to their ability to provide optimal pain relief. In trauma patients, appropriate blocks based on the localization of injury can be successfully performed by emergency physicians.

In upper extremity trauma, ESP block, clavipectoral block, and superior posterior serratus intercostal block have been effectively utilized [28]. For rib fractures, ESP and SAP blocks are commonly employed [29]. In anterior thoracic trauma, the PECS II, and in abdominal injuries, the QL block have been reported to be used successfully in the literature [30,31]. Beyond trauma, FPBs have also been used for a wide range of conditions, including cholecystitis [32], cancer-related pain [33,34], and endometriosis-associated pain [35].

## 8. Fascial Plane Blocks in Pediatric Patients

Despite growing evidence supporting their efficacy, the use of FPBs in pediatric patients remains proportionally lower compared with adults. However, this trend is changing with improved training, increasing clinician experience, reduced concerns regarding complications, and the widespread adoption of ultrasound guidance.

Although pediatric regional anesthesia techniques are often perceived as technically more challenging, the thinner muscle layers and lower adipose tissue content in children actually facilitate ultrasonographic visualization of target structures, needle advancement, and local anesthetic spread compared with adults. A critical consideration, however, is the age-related variability in anatomical and structural characteristics. In neonates and infants, fascial layers and connective tissues are looser and more elastic, allowing local anesthetic solutions to spread more easily and over a wider area between fascial planes. While this characteristic enables effective blockade with lower volumes, it may also increase the risk of systemic absorption and local anesthetic systemic toxicity (LAST) [36]. In pediatric patients, maximum local anesthetic doses should be strictly calculated on a mg/kg basis, considering age-related pharmacokinetic variability. Neonates and infants have reduced plasma protein binding and immature hepatic metabolism, potentially increasing free drug fractions and toxicity risk. Consequently, lower concentration solutions and conservative total dose limits are recommended, particularly when bilateral blocks are performed. Recommended doses for most plane blocks are 0.3–0.5 mL/kg of 0.25% bupivacaine or 0.2% ropivacaine (maximum 20 mL/kg).

Any FPB that is appropriately indicated in adult patients can, in principle, be applied in pediatric populations. Nevertheless, meticulous dose calculation based on age and body weight—ensuring that maximum recommended doses are not exceeded—is essential to prevent systemic toxicity. In contrast to adults, fascial plane blocks in pediatric patients are most commonly performed after induction of general anesthesia (either before or after the procedure), particularly in non-operating room anesthesia settings. This approach minimizes patient movement and helps prevent psychological distress.

One of the most recent and comprehensive studies in pediatric interventional radiology was the retrospective analysis by Gaelen et al. [37], which evaluated 309 pediatric patients undergoing sclerotherapy for bone cysts, venous malformations, and lymphatic malformations. In the group receiving regional anesthesia, opioid requirements were significantly reduced both intraoperatively and postoperatively. Notably, among children treated for bone cysts, opioid use during hospitalization was 62.7% in the block group compared with 100% in the non-block group (*p* < 0.001). The study demonstrated that regional blocks, when applied without causing workflow delays, significantly reduced postoperative pain and opioid consumption and represented a safe and effective component of multimodal analgesia in pediatric patients [37].

## 9. Pharmacokinetics and the Risk of LAST

With the introduction of ultrasonography into clinical practice, significant reductions in local anesthetic dose and volume have been achieved in peripheral nerve blocks (e.g., axillary block) due to direct visualization of neural structures. In contrast, fascial plane blocks represent a fundamentally different paradigm: these techniques are volume-dependent, and the extent of analgesia relies primarily on the volume of local anesthetic administered and its spread within the fascial plane. Attempts to achieve a wider and more effective blockade by increasing injected volumes inevitably raise the risk of LAST.

The pharmacokinetic properties of local anesthetics in FPBs have been well described. The rate of systemic absorption is directly related to the surface area of tissue contact and the degree of tissue vascularization. Fascial planes, characterized by large surface areas and rich vascular networks between muscle groups, are particularly prone to rapid absorption. Consequently, high-volume injections may result in a rapid rise in plasma local anesthetic concentrations.

For bupivacaine (0.25%), the recommended maximum single-dose limit generally should not exceed 2 mg/kg, with an absolute maximum of 150 mg. Ropivacaine (0.2–0.5%), owing to its more favorable cardiotoxicity profile, is frequently preferred for fascial plane blocks, with a commonly accepted maximum dose of approximately 3 mg/kg. For most unilateral fascial plane blocks (e.g., TAP, ESP, SAP), a volume of 20–30 mL is typically recommended. To allow larger volumes while maintaining safety, lower concentrations (0.125–0.25%) may be used [38].

In the event of suspected LAST, immediate management should follow established guidelines, including airway stabilization, seizure control, and early administration of 20% lipid emulsion therapy (initial bolus, 1.5 mL/kg over 1 min, followed by infusion, 0.25 mL/kg/min). When bilateral or combined fascial plane blocks are performed, cumulative dosing must be carefully calculated to avoid exceeding recommended maximum systemic doses.

## 10. Safety Considerations and Complications of Fascial Plane Blocks

The safety profile of FPBs is generally favorable, largely because neural structures are not directly targeted and ultrasound guidance allows real-time visualization of needle trajectory and local anesthetic spread. Nevertheless, given the volume-dependent nature of these techniques and their frequent application in patients with limited physiological reserve in NORA settings, structured risk mitigation strategies are essential. Table 3 summarizes the principal complications associated with fascial plane blocks, outlining their underlying mechanisms, predisposing factors, and key prevention strategies relevant to clinical practice in NORA settings.

## 11. Evidence Synthesis and Quality Considerations

The current evidence supporting the use of FPBs in NORA settings is heterogeneous. While several randomized controlled trials exist (particularly in device implantation procedures), a substantial portion of the literature consists of retrospective studies, single-center analyses, and case series.

In interventional radiology applications such as PTBD and hepatic ablation, most data derive from observational studies with limited sample sizes. Therefore, although analgesic efficacy appears promising, high-quality multicenter randomized trials remain scarce.

The strength of evidence varies by procedural category, being strongest for pectoral plane blocks in device implantation and more limited for visceral interventional procedures.

Accordingly, current conclusions should be interpreted within the context of evolving evidence rather than definitive practice-changing data.

## 12. Conclusions

In NORA settings, FPBs have become an indispensable component of modern anesthetic practice due to their ability to provide effective analgesia, low complication rates, opioid-sparing effects, and improved patient comfort. In painful procedures such as S-ICD implantation and hepatic ablation, these techniques offer a safe alternative to general anesthesia when combined with sedation. With ongoing advances in anatomical understanding, ultrasound technology, and the introduction of novel blocks (e.g., clavipectoral fascia plane block, fascia lata plane block), the scope of fascial plane blocks continues to expand. In the future, these techniques may increasingly become integrated into NORA protocols as part of a multimodal analgesic strategy; however, their role as a routine standard of care will depend on further high-quality comparative studies and procedure-specific evidence.

## Figures and Tables

**Table 1:** Thoracic fascial plane blocks used for analgesia in non-operating room anesthesia settings.

Block	Target Fascial Plane	Dermatomal/Anatomical Coverage	Typical NORA Indications	Key Advantages/Limitations	Local Anesthetic Type, Concentration, and Typical Dose *
Interpectoral (PECS I) Block	Between the pectoralis major and minor muscles	Pectoral region (motor and sensory input of pectoral muscles)	Pacemaker or ICD pocket creation, port implantation	Simple technique; limited cutaneous analgesia	Ropivacaine 0.25–0.375%, 10–15 mL
Pectoserratus (PECS II) Block	PECS I plane + between the pectoralis minor and serratus anterior muscles	Anterolateral chest wall (T2–T6)	ICD/S-ICD implantation, breast and anterior thoracic procedures	Broader coverage than interpectoral; superficial and safe	Ropivacaine 0.25–0.375%, total 20–30 mL
Serratus Anterior Plane Block (superficial or deep)	Superficial or deep to the serratus anterior muscle	Lateral thoracic wall (T2–T9)	Chest wall interventions, thoracic drains, ICD leads	Effective lateral wall analgesia; minimal sympathetic block	Ropivacaine 0.25% or Bupivacaine 0.25%, 20–30 mL
Transversus Thoracic Muscle Plane Block	Between the internal intercostal muscles and the transversus thoracis muscle	Parasternal region (T2–T6 anterior branches)	S-ICD implantation, parasternal access procedures	Targeted parasternal analgesia; technically more demanding	Ropivacaine 0.25%, 10–15 mL per side
Parasternal Intercostal Plane Block	Superficial or deep parasternal plane	Anterior chest wall (T2–T6)	Sternum-related procedures, parasternal pain	Focused cutaneous and deep analgesia	Ropivacaine 0.25%, 10–15 mL per side
Erector Spinae Plane Block	Deep to the erector spinae muscle at the transverse process	Multisegmental thoracic analgesia (T2–T10)	Extensive chest wall procedures, combined with thoracic pain	Wide dermatomal spread; simple and safe	Ropivacaine 0.25–0.375%, 20–30 mL
Retrolaminar Block	Between the lamina and the deep paraspinal muscles	Posterior thoracic wall	Posterior thoracic interventions	Alternative to ESP; less anterior spread	Ropivacaine 0.25%, 20–25 mL

* Reported doses represent commonly used ranges in the literature. Total local anesthetic dose should be individualized based on patient weight, comorbidities, and institutional safety protocols. PECS I: interpectoral block; PECS II: pectoserratus block; NORA: non-operating room anesthesia; S-ICD: subcutaneous implantable cardioverter–defibrillator.

**Table 2:** Abdominal fascial plane blocks used for analgesia in non-operating room anesthesia settings.

Block	Target Fascial Plane	Dermatomal/Anatomical Coverage	Typical NORA Indications	Key Advantages/Limitations	Local Anesthetic Type, Concentration, and Typical Dose *
Transversus Abdominis Plane (TAP) Block	Between the internal oblique and transversus abdominis muscles	Anterior abdominal wall (T6–L1, technique-dependent)	Percutaneous abdominal interventions, drain placement, biopsy	Simple and widely used; limited visceral analgesia	Ropivacaine 0.25–0.375% or Bupivacaine 0.25%, 20–30 mL per side
Subcostal TAP Block	Subcostal TAP plane	Upper abdominal wall (T6–T9)	Upper abdominal procedures, hepatic or biliary interventions	Better supraumbilical coverage	Ropivacaine 0.25–0.375%, 20–30 mL
Rectus Sheath Block	Between the rectus abdominis muscle and the posterior rectus sheath	Midline abdominal wall (T7–T12)	Umbilical or midline access procedures	Targeted midline analgesia; no lateral coverage	Ropivacaine 0.25%, 10–15 mL per side
Ilioinguinal–Iliohypogastric Block	Between the internal oblique and transversus abdominis near the anterior superior iliac spine	Lower abdominal wall, groin (L1)	Inguinal procedures, femoral access-related pain	Focused lower abdominal analgesia	Ropivacaine 0.25%, 10–15 mL per side
Quadratus Lumborum (QL) Block—Type 1 (Lateral)	Anterolateral border of QL muscle	T7–L1 (variable spread)	Extensive abdominal wall analgesia	Potential visceral analgesia; variable spread	Ropivacaine 0.25–0.375%, 20–30 mL
Quadratus Lumborum (QL) Block—Type 2 (Posterior)	Posterior to QL muscle	T7–L1, paravertebral extension	Abdominal and retroperitoneal procedures	Longer duration; technically more demanding	Ropivacaine 0.25–0.375%, 20–30 mL
Transmuscular QL Block (QL3)	Between the QL and psoas major muscles	T6–L1, possible visceral analgesia	Major abdominal interventions	Widespread; proximity to the lumbar plexus	Ropivacaine 0.25–0.375%, 20–30 mL
Abdominal Erector Spinae Plane (ESP) Block	Deep to the erector spinae muscle at the lower thoracic/lumbar level	Multisegmental abdominal wall analgesia	Extensive abdominal procedures, opioid-sparing strategies	Simple and safe; indirect spread	Ropivacaine 0.25–0.375%, 20–30 mL

* Reported doses represent commonly used ranges in the literature. Total local anesthetic dose should be individualized based on patient weight, comorbidities, and institutional safety protocols. NORA: non-operating room anesthesia; TAP: transversus abdominis plane; QL: quadratus lumborum; ESP: erector spinae plane.

**Table 3:** Mechanisms, risk factors, and prevention strategies for complications associated with fascial plane blocks.

Complication	Mechanism	Predisposing Factors	Prevention Strategy
LAST	Rapid systemic absorption from vascular fascial planes	High total dose, bilateral blocks, low body weight	Weight-based dosing, incremental injection, ultrasound guidance, lipid rescue availability
Pneumothorax	Pleural puncture during thoracic blocks	Deep needle trajectory, inadequate visualization	Continuous needle-tip visualization, in-plane technique
Hematoma	Vascular injury	Anticoagulation, deep posterior blocks	Pre-procedural coagulation assessment, ultrasound Doppler use
Unintended neuraxial spread	Paravertebral/epidural diffusion	Posterior approaches (QL, ESP)	Controlled-volume injection, monitoring hemodynamics
Infection	Breach of sterile technique	Prolonged procedure, immunosuppression	Strict aseptic precautions

LAST: local anesthetic systemic toxicity; QL: quadratus lumborum; ESP: erector spinae plane.

## Data Availability

No new data were created or analyzed in this study.

## Abbreviations

NORA	Non-operating room anesthesia
FPBs	Fascial plane blocks
MRI	Magnetic resonance imaging
CT	Computed tomography
ESP	Erector spinae plane
TTMP	transversus thoracic muscle plane TTMP
S-ICD	Subcutaneous implantable cardioverter–defibrillator
SAP	Serratus anterior plane
TAP	Transversus abdominis plane
QL	Quadratus lumborum
ICD	Implantable cardioverter–defibrillator
CPFB	clavipectoral fascial plane block
RFA	Radiofrequency ablation
PTBD	Percutaneous transhepatic biliary drainage
NRS	Numeric Rating Scale
EVAR	Endovascular aortic repair
TEVAR	Thoracic endovascular aortic repair
TAVI	Transcatheter aortic valve implantation
LAST	Local anesthetic systemic toxicity
